# Long non-coding and coding RNA profiling using strand-specific RNA-seq in human hypertrophic cardiomyopathy

**DOI:** 10.1038/s41597-019-0094-6

**Published:** 2019-06-13

**Authors:** Xuanyu Liu, Yi Ma, Kunlun Yin, Wenke Li, Wen Chen, Yujing Zhang, Changsheng Zhu, Tianjiao Li, Bianmei Han, Xuewen Liu, Shuiyun Wang, Zhou Zhou

**Affiliations:** 1 Center of Laboratory Medicine, Fuwai Hospital, State Key Laboratory of Cardiovascular Disease, Beijing Key Laboratory for Molecular Diagnostics of Cardiovascular Diseases, National Center for Cardiovascular Diseases, Chinese Academy of Medical Sciences and Peking Union Medical College, Beijing, 100037 China; 2 Departments of Cardiovascular Surgery, Fuwai Hospital, State Key Laboratory of Cardiovascular Disease, National Center for Cardiovascular Diseases, Chinese Academy of Medical Sciences and Peking Union Medical College, Beijing, 100037 China

**Keywords:** Gene expression analysis, Cardiac hypertrophy

## Abstract

Hypertrophic cardiomyopathy (HCM) represents one of the most common heritable heart diseases. However, the signalling pathways and regulatory networks underlying the pathogenesis of HCM remain largely unknown. Here, we present a strand-specific RNA-seq dataset for both coding and lncRNA profiling in myocardial tissues from 28 HCM patients and 9 healthy donors. This dataset constitutes a valuable resource for the community to examine the dysregulated coding and lncRNA genes in HCM versus normal conditions.

## Abstract (summary)

**Table:** 

Design Type(s)	transcription profiling design • disease state design • sequence analysis objective
Measurement Type(s)	transcription profiling assay
Technology Type(s)	RNA sequencing
Factor Type(s)	experimental condition
Sample Characteristic(s)	Homo sapiens • heart

Machine-accessible metadata file describing the reported data (ISA-Tab format)

## Background & Summary

Hypertrophic cardiomyopathy (HCM) represents one of the most common heart diseases (an estimated prevalence of at least 0.2%), and a leading cause of sudden death in young people^1,2^. HCM is generally regarded as a genetic disorder caused predominately by mutations in eight sarcomere genes, including *MYH7*, *MYBPC3*, *ACTC1*, *TPM1*, *MYL2*, *MYL3*, *TNNI3*, and *TNNT2*^3^. However, the signalling pathways and regulatory networks underlying the pathogenesis of HCM remain largely unknown.

Long non-coding RNAs (lncRNAs) are a large class of transcripts ≥200 nucleotides in length that do not encode proteins^4^. Compared with coding mRNAs, most lncRNAs are less well annotated and their functions are largely unexplored. Nevertheless, there is increasing evidence showing that lncRNAs are involved in a variety of biological processes and diseases^5,6^. lncRNAs have been implicated in pathologically processes of HCM, such as cardiomyocyte disarrangement, myocardial hypertrophy and interstitial fibrosis^7^. Through comparative analysis between 7 HCM patients and 5 control subjects using microarray, dysregulated lncRNAs in myocardial tissues of HCM patients were found to be involved in the pathogenesis of HCM through the regulation of pathogenetic pathways^8^.

RNA-seq is emerging as the major transcriptome profiling system. RNA-seq has considerable advantages over microarray in many aspects such as novel transcript identification through de novo assembly, splice junction identification and allele-specific expression analysis. Compared with the standard RNA-seq protocol, strand-specific RNA-seq retains strand of origin information, thus providing a greater resolution for sense/antisense profiling, which is essential for antisense lncRNA identification^9^. To our knowledge, we still lack a strand-specific RNA-seq dataset for myocardial tissues of HCM patients.

Here, we present a strand-specific RNA-seq dataset for both coding and lncRNA profiling in myocardial tissues from 28 HCM patients and 9 healthy donors. This dataset constitutes a valuable resource for the community to examine the dysregulated coding and lncRNA genes in HCM versus normal conditions. This dataset may also be reutilized through integration with future datasets to further enhance statistical power by increasing sample size.

## Methods

### Ethical approval

This study was approved by the ethics committee of the institutional review board at Fuwai Hospital. All procedures were conducted according to the ethical standards of the research committee. Informed consent was obtained from all subjects.

### Patients and sample collection

**Fig. 1:**
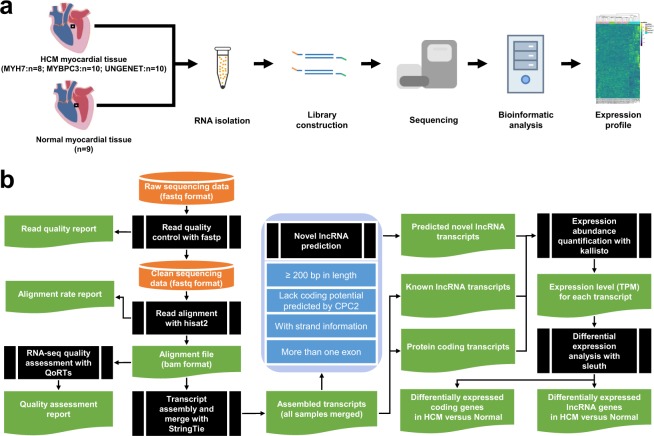
Overview of the experimental procedure. (**a**) Schematic representation of the experimental workflow. The sampling position is indicated by a black rectangular. RNA isolation and library preparation for all samples were performed in the same batch. HCM: hypertrophic cardiomyopathy; GENETUN: Genetically undiagnosed HCM; MYBPC3: HCM patient with mutation in *MYBPC3*; MYH7: HCM patient with mutation in *MYH7*; NORMAL: Normal heart. (**b**) Bioinformatic analysis workflow.

Figure 1a shows the experimental design and workflow. We enrolled 28 HCM patients undergoing septal myectomy (Table 1 and Supplementary Table S1). All these patients were subjected to genetic testing to identify pathogenic mutations in HCM causal genes. Among them, 10 patients were confirmed to carry deleterious mutation in the gene *MYBPC3* encoding cardiac myosin binding protein C, and 8 patients in the gene *MYH7* encoding beta-myosin heavy chain. The remaining 10 patients were genetically undiagnosed. Myocardial tissues were collected during surgery and immediately placed in liquid nitrogen for storage. In addition, myocardial tissues in a normal, healthy condition (9 samples as a control group) were collected from donor hearts abandoned during cardiac transplant due to unexpected reasons.

**Table 1:** Summary statistics for the sequencing data.

Sample	Group	#read pairs	#bases (G)	Q20	Q30	overall alignment rate
HCM269	GENETUN	51,524,526	14.35	97.89%	94.49%	97.79%
HCM273	GENETUN	49,447,308	13.77	97.88%	94.50%	97.93%
HCM282	GENETUN	66,813,232	18.61	98.30%	95.44%	98.00%
HCM395	GENETUN	49,855,174	13.88	97.77%	94.27%	97.27%
HCM405	GENETUN	47,625,057	13.27	97.85%	94.42%	97.70%
HCM420	GENETUN	57,763,729	16.08	97.86%	94.42%	97.76%
HCM493	GENETUN	66,750,733	18.55	97.73%	94.15%	97.54%
HCM541	GENETUN	58,387,469	16.29	98.27%	95.33%	98.13%
HCM552	GENETUN	51,288,461	14.29	97.77%	94.25%	97.48%
HCM591	GENETUN	57,727,967	16.07	98.06%	94.92%	97.74%
HCM439	MYBPC3	57,023,162	15.87	97.76%	94.20%	97.57%
HCM460	MYBPC3	41,034,383	11.42	97.82%	94.32%	97.71%
HCM486	MYBPC3	50,228,447	13.99	97.80%	94.27%	97.79%
HCM498	MYBPC3	58,077,468	16.15	97.84%	94.43%	97.40%
HCM504	MYBPC3	62,460,847	17.41	98.27%	95.34%	98.11%
HCM515	MYBPC3	62,410,260	17.38	98.31%	95.45%	98.00%
HCM518	MYBPC3	60,673,907	16.89	98.01%	94.81%	97.62%
HCM533	MYBPC3	56,061,058	15.60	97.83%	94.42%	97.63%
HCM429	MYBPC3	52,807,037	14.71	97.64%	93.95%	97.60%
HCM437	MYBPC3	56,355,435	15.71	97.83%	94.39%	97.66%
HCM431	MYH7	47,270,654	13.15	98.55%	95.97%	97.87%
HCM443	MYH7	50,878,232	14.16	97.80%	94.28%	97.49%
HCM456	MYH7	61,153,662	17.01	97.85%	94.39%	97.78%
HCM483	MYH7	65,366,081	18.19	98.32%	95.47%	97.97%
HCM490	MYH7	53,694,284	14.94	97.78%	94.29%	97.65%
HCM491	MYH7	60,649,986	16.87	97.80%	94.33%	97.58%
HCM506	MYH7	51,473,866	14.31	97.86%	94.39%	97.74%
HCM562	MYH7	58,882,347	16.37	98.37%	95.59%	98.03%
N102-LV	NORMAL	54,725,491	15.25	97.43%	93.70%	97.59%
N103-LV	NORMAL	72,263,796	20.14	98.26%	95.33%	97.99%
N104-LV	NORMAL	74,732,382	20.77	98.26%	95.33%	98.06%
N105-LV	NORMAL	61,657,432	17.15	98.30%	95.39%	98.06%
ND1-LV	NORMAL	54,854,093	15.25	98.41%	95.63%	98.13%
ND2	NORMAL	57,230,198	15.90	98.34%	95.49%	97.86%
sc2-LV	NORMAL	59,025,988	16.41	98.34%	95.49%	98.16%
sc5-LV	NORMAL	56,871,247	15.84	98.32%	95.48%	97.67%
sc6-LV	NORMAL	65,688,425	18.27	98.46%	95.76%	97.87%

GENETUN: Genetically undiagnosed HCM patient; MYBPC3: HCM patient with mutation in *MYBPC3*; MYH7: HCM patient with mutation in *MYH7*; NORMAL: Normal heart.

### RNA isolation and qualification

Total RNA was isolated with TRIzol^TM^ reagent (Invitrogen, USA) according to the manufacturer’s instruction. RNA concentration was measured using Qubit^®^ RNA Assay Kit in Qubit^®^ 2.0 Fluorometer (Life Technologies, CA, USA). RNA purity was assessed using the NanoPhotometer^®^ spectrophotometer (IMPLEN, CA, USA). RNA integrity was checked using the RNA Nano 6000 Assay Kit on the Agilent Bioanalyzer 2100 system (Agilent Technologies, CA, USA). Only samples with a 260:280 ratio of ≥1.5 and an RNA integrity number (RIN) of ≥8 were subjected to deep sequencing.

### Strand-specific RNA-seq library preparation & sequencing

We prepared a strand-specific RNA-seq library for each sample. Firstly, ribosomal RNA (rRNA) was removed by Epicentre Ribo-zero^TM^ rRNA Removal Kit (Epicentre, USA) from 3 μg total RNA. Then, sequencing libraries were generated using NEBNext^®^ Ultra^TM^ Directional RNA Library Prep Kit for Illumina^®^ (NEB, USA) following manufacturer’s instructions. Briefly, the first strand cDNA synthesis was performed using M-MuLV reverse transcriptase and random hexamer primer. The second strand cDNA was synthesized using RNase H and DNA Polymerase I. The dTTP was replaced by dUTP in the reaction buffer. Following end repair and adenylation, cDNA fragments were ligated to adaptors. Then, 3 μl USER Enzyme was incubated with the cDNA for 15 min at 37 °C followed by 5 min at 95 °C before PCR. Following PCR amplification, products were purified using the AMPure XP system. Finally, library quality was assessed on the Agilent Bioanalyzer 2100 system. The resulting libraries were sequenced on the Illumina HiSeq X Ten System in a 2 × 150 bp paired-end mode.

### Read alignment and transcript assembly

Figure 1b shows the bioinformatic analysis workflow. The raw sequencing reads^10^ were subjected to adapter trimming and base quality filtering by fastp v0.7.0^11^. Clean reads obtained were aligned to the human reference genome (GRCh37) using hisat2 v2.1.0^12^ under default settings. Following alignment, the quality of each RNA-seq dataset was assessed through a variety of metrics generated by QoRTs^13^. Transcript de novo assembly for each sample was performed using StringTie v1.3.4b^14^ under default settings with the guidance of a reference annotation (GENCODE GRCh37 release 27, -G option). The assembled transcripts of all samples were merged into a single file using the merge function of StringTie with the reference annotation provided (-G option). Other parameters were set to defaults (-m 50 -T 1 -f 0.01 -g 250).

### Novel lncRNA gene prediction

The transcripts without matched known transcript information in the StringTie merge output were predicted to be from novel lncRNA genes based on the following criteria: (1) the novel transcripts assembled must have definite strand information; (2) the transcripts must have more than one exon; (3) the transcripts must be more or equal to 200 bp in length; and (4) the coding potential of the transcripts were predicted using CPC2^15^, and only the transcripts labelled as “noncoding” in the output were kept. We ultimately got 205 novel lncRNA genes (ALL_GENE_EXPR_DEG_ANALYSIS.xlsx)^16^.

### Expression abundance quantification

**Fig. 2:**
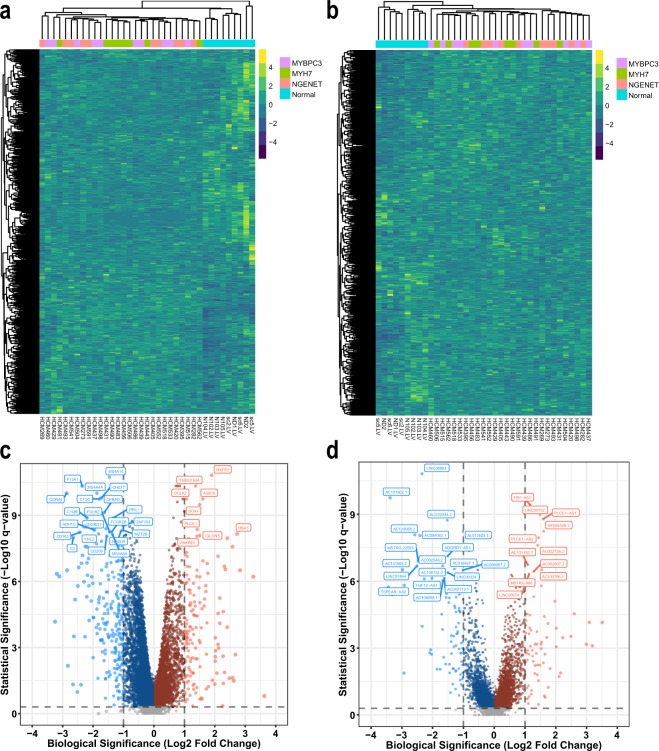
Expression profiles of coding and lncRNA genes. (**a**) Hierarchical clustering of the samples from the three HCM groups and the normal group based on the expression of coding genes. (**b**) Hierarchical clustering of the samples from the three HCM groups and the normal group based on the expression of lncRNA genes. In a and b, each row represents a gene, and each column represents a sample. For better visualization, only the expression of 1,000 randomly selected genes are displayed on the heatmap. (**c**) Volcano plot showing the differentially expressed coding genes between HCM and normal groups. (**d**) Volcano plot showing the differentially expressed lncRNA genes between HCM and normal groups. In c and d, dots coloured in light red or light blue denote statistically and biologically significant genes being up-regulated or down-regulated, respectively. The dot size reflects the absolute fold change. Only the top 30 DEGs were labelled with gene symbols.

All coding genes and lncRNA genes, including predicted novel lncRNA, lincRNA, sense intronic lncRNA, sense overlapping lncRNA and antisense lncRNA genes, were incorporated in expression abundance quantification (stringtie_merged.strand.lncRNA.proteincoding.gtf)^16^. Firstly, the transcript sequences (stringtie_merged.strand.lncRNA.proteincoding.fa)^16^ were extracted from the reference genome using gffread (https://github.com/gpertea/gffread). Then, the expression of the transcripts was quantified with kallisto v0.43.1^17^ under default settings. For comparison among samples, transcript abundance for each sample was normalized with Transcripts Per Million (TPM)^18^. The expression of each gene was determined by aggregating the expression of all corresponding transcript isoforms. Along with transcript abundance estimates, 100 bootstraps per sample were generated (kallisto quant –b 100), which serve as proxies for technical replicates. Figure 2a,b show the expression profiles of coding genes and lncRNA genes in each sample, respectively. Based on the expression of coding genes, hierarchical clustering analysis revealed distinct expression landscapes between the normal and HCM groups for both coding and lncRNA genes. However, samples from each of the three HCM groups were not clustered together, indicating that there may be no significant difference in transcriptome among HCM patients with different genetic backgrounds at least in the sampling stage.

### Differential expression analysis

Following quantification, the identification of differentially expressed genes (DEGs) between HCM and normal samples was performed using sleuth v0.29.0^19^, which could leverage the bootstraps of kallisto to correct for technical variation. The biological significance threshold was set to a fold change of ±2 fold, and the statistical significance threshold was set to a q-value of 0.05 (−log10 q-value > 1.3). Only genes that achieved both biological and statistical significance were considered as DEGs. We identified 132 and 241 coding genes up-regulated and down-regulated in HCM versus normal samples, respectively (Fig. 2c). We also found 67 and 83 lncRNA genes up-regulated and down-regulated in HCM versus normal samples, respectively (Fig. 2d). We made available the useful information for each sample, including the expression abundance of each gene, testing statistics and DEGs (ALL_GENE_EXPR_DEG_ANALYSIS.xlsx)^16^.

## Data Records

The sequencing data in the fastq format have been deposited in NCBI Sequence Read Archive (SRA)^10^. The transcript abundance file for each sample has been deposited in Gene Expression Omnibus (GEO)^18^. Other processed files were uploaded to figshare^16^.

## Technical Validation

After quality control, the number of sequenced bases was over 11 Gb in all samples, and the Q20 (the percentage of bases with Phred-scaled quality score ≥20) was over 97% in all samples (Q30 over 93%), indicating that the base quality was sufficiently high for downstream analyses (Table 1). When aligning the clean reads to human reference genome, the overall alignment rate was high (over 97%) in all samples, suggesting little contamination from microorganisms (Table 1).

**Fig. 3:**
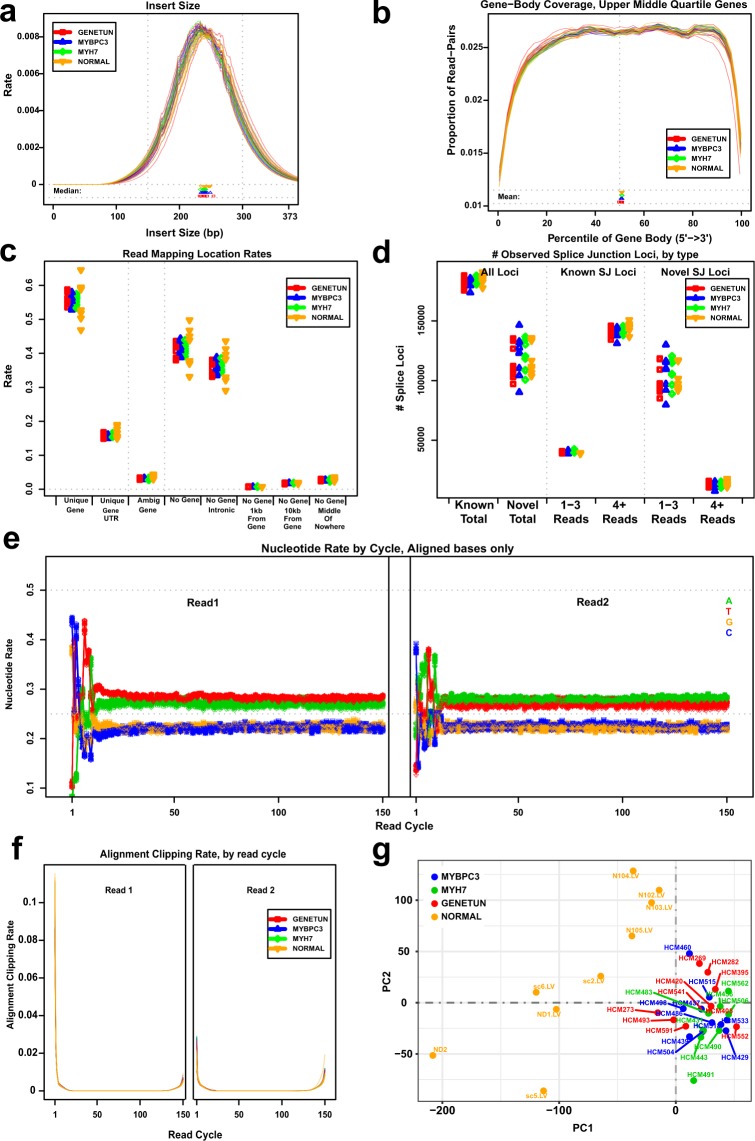
Quality assessment of the RNA-seq dataset. (**a**) Distribution curve of estimated insert size for each sample. (**b**) Gene body coverage profile for each sample. Only the genes in the upper-middle quartile by read-count are considered. (**c**) Read mapping rates for different location categories in each sample. Unique Gene: exons of only one gene; Unique Gene UTR: UTRs of only one gene; Ambig Gene: exons of more than one gene; No Gene: a region without annotated genes; No Gene, Intronic: a region bridged by an annotated splice junction; No gene, 1 kb from gene: 1 kilobase from the nearest annotated gene; No gene, 10 kb from gene: 10 kilobases from the nearest annotated gene; No gene, middle of nowhere: more than 10 kilobases from the nearest annotated gene (**d**) Number of splice junctions for different categories of each sample. “1–3 reads” means the junction locus is covered by 1–3 read-pairs. (**e**) Nucleotide rate by cycle for aligned bases in each sample. Nucleotide types are differentiated by colour. Sample groups are differentiated by shape. (**f**) Alignment soft clipping rate by cycle in each sample. (**a**–**f**) Plots are generated by QoRTs. (**g**) PCA for visualizing the high-dimensional expression datasets.

Taking advantage of QoRTs^13^, a toolkit for quality assessment of RNA-seq dataset, we made cross-comparisons of samples to identify any outliers or systematic errors associated with biological conditions, i.e., different groups (Fig. 3a–f). Figure 3a shows the distribution curve of estimated insert size for each sample. We found that the curves were relatively smooth (no “spikes”) and consistent across samples and conditions, reflecting little technical bias across samples. Figure 3b shows the gene body coverage profile for each sample, and no significant 3’ bias was found, indicating that the datasets were not affected by RNA degradation. Figure 3c shows the read mapping rates for different location categories in each sample, from which we did not observe any outlier within each condition, suggesting consistency across samples in terms of alignment. Similarly, we did not observe a disproportionate identification of novel splice junctions in one sample or condition (Fig. 3d). Except for the nucleotide composition bias in the first few cycles that normally occur in Illumina RNA-seq data, the base composition was quite uniform across all other cycles (Fig. 3e). Figure 3f shows the alignment soft clipping rate by cycle in each sample. We did not observe any “spikes” in the curves for all samples and the clipping profiles were generally consistent across samples and conditions. To visualize the high-dimensional transcriptomic datasets, we performed dimension reduction with principle component analysis (PCA). Consistent with the observation in the hierarchical clustering analysis (Fig. 2a,b), we found that all HCM samples clustered together and were distant from normal samples (Fig. 3g), thus suggesting that our data are suitable for differential expression analysis. As expected, the transcriptomic variance among samples was found to be more significant in the normal condition than the diseased HCM condition.

Taken together, we presented a high-quality dataset that was suitable for differential expression and splicing analysis of both coding and lncRNA genes in myocardial tissues between HCM and normal conditions.

## Supplementary Information

### ISA-Tab metadata file

Download metadata file

### Supplementary Information

Table S1

## Data Availability

The code for processing the data from raw sequencing reads to DEGs is available within figshare (CODE_for_RNA-seq.sh)^16^.
